# Inflammasomes in Teleosts: Structures and Mechanisms That Induce Pyroptosis during Bacterial Infection

**DOI:** 10.3390/ijms22094389

**Published:** 2021-04-22

**Authors:** Natsuki Morimoto, Tomoya Kono, Masahiro Sakai, Jun-ichi Hikima

**Affiliations:** 1 Interdisciplinary Graduate School of Agriculture and Engineering, University of Miyazaki, 1-1 Gakuenkibanadai-nishi, Miyazaki 889-2192, Japan; nmorimoto0902@gmail.com; 2 Department of Biochemistry and Applied Bioscience, Faculty of Agriculture, University of Miyazaki, 1-1 Gakuenkibanadai-nishi, Miyazaki 889-2192, Japan; tkono@cc.miyazaki-u.ac.jp (T.K.); m.sakai@cc.miyazaki-u.ac.jp (M.S.)

**Keywords:** pattern recognition, pyroptosis, inflammasome, NLR, ASC, Caspase-1, gasdermin

## Abstract

Pattern recognition receptors (PRRs) play a crucial role in inducing inflammatory responses; they recognize pathogen-associated molecular patterns, damage-associated molecular patterns, and environmental factors. Nucleotide-binding oligomerization domain-leucine-rich repeat-containing receptors (NLRs) are part of the PRR family; they form a large multiple-protein complex called the inflammasome in the cytosol. In mammals, the inflammasome consists of an NLR, used as a sensor molecule, apoptosis-associated speck-like protein containing a caspase recruitment domain (ASC) as an adaptor protein, and pro-caspase1 (Casp1). Inflammasome activation induces Casp1 activation, promoting the maturation of proinflammatory cytokines, such as interleukin (IL)-1β and IL-18, and the induction of inflammatory cell death called pyroptosis via gasdermin D cleavage in mammals. Inflammasome activation and pyroptosis in mammals play important roles in protecting the host from pathogen infection. Recently, numerous inflammasome-related genes in teleosts have been identified, and their conservation and/or differentiation between their expression in mammals and teleosts have also been elucidated. In this review, we summarize the current knowledge of the molecular structure and machinery of the inflammasomes and the ASC-spec to induce pyroptosis; moreover, we explore the protective role of the inflammasome against pathogenic infection in teleosts.

## 1. Introduction

Inflammatory responses play a crucial role in the innate immune system to protect the host body from pathogens or other environmental factors. Pattern recognition systems act as inducers of inflammation [1,2]. For activation of the pattern recognition system, pattern recognition receptors (PRRs) recognize pathogen-associated molecular patterns (PAMPs) (e.g., lipopolysaccharide (LPS) and flagellin), damage-associated molecular patterns (DAMPs) (e.g., high mobility group box 1 (HMGB1), adenosine triphosphate (ATP)), or even environmental factors (e.g., particulate silica [3] and aluminum salt [4]) [1,2,5,6]. In the host cell cytosol, nucleotide-binding oligomerization domain (NOD)-leucine-rich repeats (LRR)-containing receptors (NLRs), part of the PRR family, recognize PAMPs, DAMPs, and environmental factors while forming a multiple-protein complex, called the “inflammasome” [1,7,8,9]. The formation and activation of the inflammasomes induce precursor inflammatory caspases (Casps), such as pro-Casp1, that activate Casp1, which then leads to non-apoptotic cell death, called “pyroptosis” [10,11,12,13]. Moreover, Casp1 induces pyroptosis and triggers precursor inflammatory cytokines to activate interleukin (IL)-1β and IL-18 [13,14,15]. These cytokines are secreted into the extracellular space through the pores formed after pyroptosis; furthermore, inflammation protected the host body from pathogens and environmental factors [14,15,16]. In this review, we discuss the activation of the inflammasome and the induction of pyroptosis in teleosts; moreover, we emphasize the potential of inflammasome activation against pathogens and environmental factors.

## 2. Inflammasome Activation to Induce Pyroptosis

### 2.1. Generic Structure of Inflammasome and Mechanisms of Pyroptosis

The inflammasome consists of an NLR (as a sensor protein), an ASC (apoptosis-associated speck-like protein containing a caspase recruitment domain (CARD), also known as pycard or the target of methylation-induced silencing-1 (TMS-1) as an adaptor protein), and pro-Casp1 (an inflammatory caspase) [16,17]. Inflammasome-forming NLRs have an N-terminal effector domain (i.e., pyrin domain (PYD) or CARD) that binds to ASC, a central fish-specific NACHT-associated domain and NACHT domain, and a C-terminal LRR motif that recognizes PAMPs, DAMPs, or environmental factors as ligands [6,9]. Several NLRs recruit the adaptor protein ASC (containing PYD and CARD) and pro-Casp1 to form a multiple-protein complex [16,17,18,19]. After recruitment of ASC and pro-Casp1, pro-Casp1 is self-proteolyzed into its active-form, Casp1 [13]. Casp1 is one of the proteases that cleaves the precursor inflammatory cytokines (i.e., pro-IL-1β and pro-IL-18) into their activated forms IL-1β and IL-18 [13,14,20]. Casp1 also cleaves gasdermin (GSDM) family proteins (i.e., GSDMA, -C, -D, and -E) at the N-terminus of GSDM, which then forms a pore in the cell membrane. Extracellular water then flows into the cell through the GSDM pore; thus, the cell expands. Eventually, the cell membrane is broken, leading to lytic cell death, known as pyroptosis [21,22]; moreover, at this point, cytosolic inflammatory molecules (including IL-1β, IL-18, HMGB1, and ATP) are released into the extracellular space [10,23,24,25]. In mammals, GSDMD induces pyroptosis in a Casp1-dependent manner [26]; however, there is no *gsdmd* in the fish genome. Current reports demonstrate that GSDME plays a key role in fish pyroptosis, as GSDMD does in mammals [27,28,29]. In zebrafish, CaspA (also known as CaspyA and Caspy) and CaspB (also known as CaspyB and Caspy2) [30], which are orthologs of Casp1, cleave the proinflammatory cytokines and two GSDMEs [27,29].

### 2.2. The Composition for Inflammasome and Apoptosis-Associated Speck-Like Protein Containing a Caspase Recruitment Domain Formation

#### 2.2.1. Inflammasome Formation

**Figure 1:**
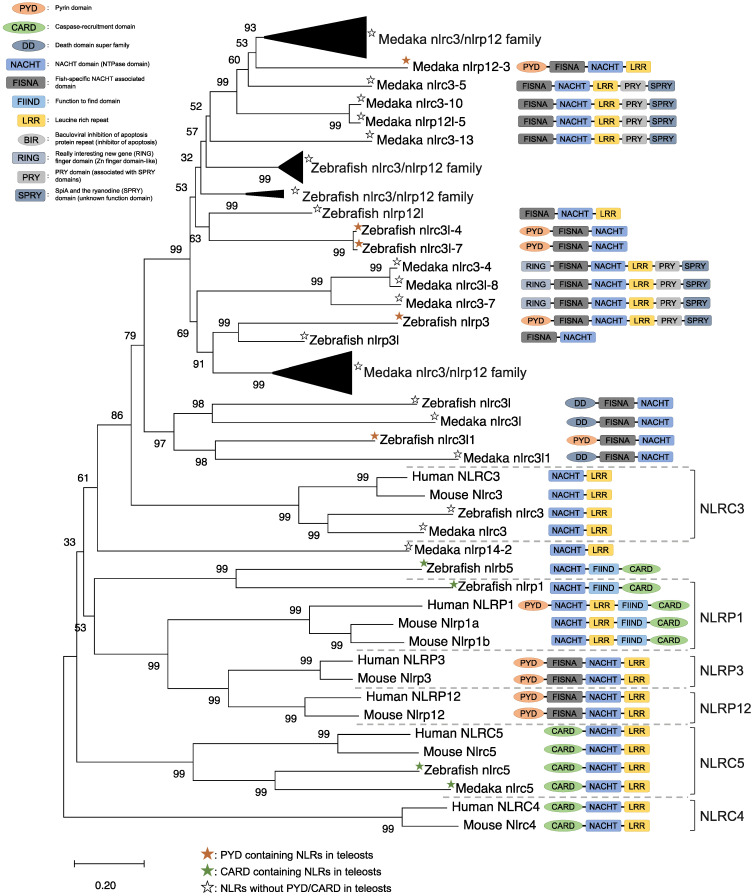
Phylogenetic relationship of the nucleotide-binding oligomerization domain-leucine-rich repeat-containing receptor family in mammals (human and mouse) and fish (Japanese medaka and zebrafish). The tree was constructed using the MEGAX and a neighbor-joining method with 1000 bootstrap replications. The black triangle shapes show collection of the clusters.

NLRs are components of the inflammasome, and they play a crucial role in recognizing PAMPs, DAMPs, and environmental factors [9]. In mammals, NLRs containing PYD (NLRP1), NLRP3, NLRP12, and CARD (NLRC4) are key sensory molecules in inflammasomes [13,31,32,33,34]. However, in teleosts, only NLRP1 and NLRP3 in zebrafish and NLRP3 in the Japanese flounder have been identified as inflammasome-forming NLRs [27,35,36]. Interestingly, Japanese medaka and zebrafish have many NLRs that belong to NLRC3 and NLRP12 families (67 in Japanese medaka and 43 in zebrafish) in the genome database, although most lack PYD or CARD, which are important in inflammasome formation [37] (Figure 1). In the phylogenetic analysis, Japanese medaka NLRP12 (NLRP12 -3), zebrafish NLRP3, and three zebrafish NLRC3-like (i.e., NLRC3-like 1, NLRC3-4, and NLRC3-7) have PYD, which is divergent from the teleost NLRC3 group (Figure 1). Furthermore, in mammals, NLRC3 consists of only NACHT and LRR. However, mammals also have PYD, DD, FISNA, RING, PRY, and SPRY, in addition to the NACHT and LRR found in the Japanese medaka and NLRC3 and NLRP12 families found in zebrafish, which have branched from the mammalian NLRC3 group.

In mice, NLRC3 inhibits TLR signaling by suppressing the signaling adaptor of TRAF6 alongside the transcription factor NF-κB [38]. However, NLRP3, which is clustered with both the zebrafish and Japanese medaka NLRC3 and NLRP12 families (Figure 1), activates the inflammatory response as an inflammasome in zebrafish. Additionally, the Japanese flounder NLRC3 involved extracellular ATP-mediated inflammatory responses [39], while Nile tilapia NLRC3 induced NF-κB activity in mammalian cells [40]. Therefore, both the NLRC3 and NLRP12 families in teleosts, which have branched from mammalian NLRC3 (Figure 1), may undergo a variety of unique evolutionary paths and have different functions from mammalian NLRCs. Furthermore, some NLRC3, NLRP12, and NLRP3 in the Japanese medaka and zebrafish conserve PRY and SPRY domains (also known as B30.2 domain), whereas these domains are not conserved in mammalian NLRs [37] (Figure 1). In zebrafish NLRP3, the B30.2 domain did not influence CaspA or CaspB activities; however, both PYD and NACHT influenced CaspA or CaspB activities [27]. According to the domain structures and phylogenetic analysis, there could be uniquely evolved inflammasome-forming NLRs with similar functions in mammals.

**Figure 2:**
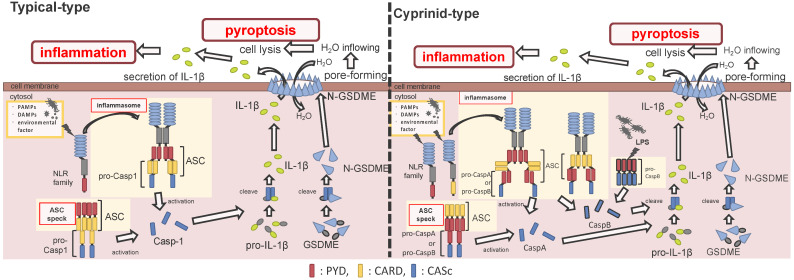
Differences between the cyprinids and other fish species in their inflammasome activation and pyroptosis pathways. In the most fish, the nucleotide-binding oligomerization domain-leucine-rich repeat-containing receptor (NLR) family recognizes the ligands, and bind to apoptosis-associated speck-like protein containing a caspase recruitment domain (ASC) and pro-Caspase1 (Casp1) via pyrin domain (PYD)-PYD and caspase recruitment domain (CARD)-CARD interactions, thus forming the inflammasome. However, in cyprinids, ligands recognize the NLR family and construct two types of inflammasomes: in the first, the NLR family binds to ASC and pro-CaspA/B via CARD–CARD and PYD–PYD interactions, respectively. The NLR family interacts with ASC via the PYD–PYD interaction, and the ASC oligomerization occurs via CARD–CARD (called ASC core). Then, the pro-CaspA/B binds to the ASC core via the PYD–PYD interaction. After activation, the Casp1/A/B undergoes self-proteolysis, and the Casp1 matures interleukin-1β, inducing pyroptosis through gasdermin-E cleavage in all fish, including cyprinids. In addition, the cyprinid’s CaspB works as a non-canonical inflammasome activator, similar to mammalian Casp4/5/11. The pro-CaspB directly recognizes liposaccharides and thus activates by itself.

ASC is an adaptor protein of the inflammasome, which is recruited after NLRs recognize ligands (Figure 2). The ASC has two functional domains, PYD and CARD, and has been identified in many fish species, including zebrafish and Japanese medaka [41,42,43,44,45,46,47]. ASC binds to NLRPs via PYD–PYD interactions in mammals [48]. Moreover, in zebrafish and Japanese flounder, NLRP3 and ASC are co-localized in the cytosol, and they are detected as small spot signals under a microscope [27,36]. Furthermore, deletion of PYD in the ASC is not co-localized with NLRP3, and CARD deletion does not influence co-localization in either zebrafish or Japanese flounder [27,36]. Therefore, NLRP3 may bind to ASC through PYD–PYD interactions in teleosts and mammals. NLRP1 has two functional domains in humans: the N-terminal PYD and C-terminal CARD domains, which are important in inflammasome formation (Figure 2). However, zebrafish NLRP1 only has a C-terminal CARD, similar to mice [31,35] (Figure 1). The N-terminal PYD of NLRP1 has autolytic activity in function to find domain (FIIND), and it inhibits the formation of the NLRP1 inflammasome in humans [31,32]. As a result, the important inflammasome-forming domain in humans, like in mice, is CARD. In mammals, NLRP1 can recruit ASC pro-Casp1 via CARD–CARD interactions [31] (Figure 2). In contrast, zebrafish NLRP1 recruits only ASC via the CARD–CARD interaction; subsequently, the ASC recruits pro-CaspA or pro-CaspB [35] (Figure 2). The difference in the NLRP1 recruitment molecules between mammals and zebrafish is thought to be related to the structure of Casp1. The differences in the structure of the aforementioned Casp1 proteins are described in the following section. In zebrafish, NLRP1 is co-localized with ASC during spot formation in the cytosol. However, the PYD-deleted ASC is not entirely co-localized with NLRP3 and oligomerizes during filament formation. In contrast, the CARD-deleted ASC does not show any co-localization with NLRP1 in zebrafish [35]. Consequently, zebrafish NLRP1 may bind to ASC via a CARD–CARD interaction (Figure 2). In a recent study, there were three replicated ASCs in Japanese medaka, all of which had PYD and CARD [42]. It is unclear whether these ASCs also bind to NLRP family members, which needs to be investigated in the future.

After the interaction between NLRs and ASC, ASC then recruits pro-Casp1, and together, they form the inflammasome (Figure 2). In all vertebrates, excluding cyprinid fish, pro-Casp1 has two functional domains, CARD and caspase consensus (CASc) (Table 1). Pro-Casp1 is self-proteolyzed into the active-form Casp1 after it binds with ASC, mutually catalyzing other pro-Casp1s [49,50]. Japanese flounder pro-Casp1 was also activated by NLRP3 [36]. In contrast, cyprinids pro-CaspA and pro-CaspyB possess PYD and CASc domains instead of CARD; moreover, zebrafish pro-CaspA and-B are co-localized with NLRP1, NLRP3, and ASC in the spot formation [27,35]. Therefore, in teleosts, pro-Casp1 is activated via NLRP1 or NLRP3 inflammasome formation.

To determine the similarity between ASC-binding domains in the vertebrate Casp1 families, the CARD domains of typical Casp1s were compared to the PYD domain in the cyprinid-types (Figure S1). In the alignment and WebLogo analyses, 41 amino acid residues (Met^1^, Ala^2^, Asp^3^, Lys^7^, Leu^9^, Arg^13^, Phe^16^, Val^20^, Ile^25^, Leu^28^, Leu^29^, Asp^30^, Leu^32^, Leu^33^, Glu^34^, Val^37^, Leu^38^, Asn^39^, Glu^42^, Glu^44^, Glu^50^, Asn^51^, Asp^56^, Ala^58^, Arg^59^, Leu^61^, Ile^62^, Asp^63^, Val^65^, Lys^68^, Gly^69^, Ala^72^, Ile^77^, Asp^84^, Leu^87^, Leu^91^, Gly^92^, Leu^93^, Thr^95^, His^96^, and Ile^97^) in the general teleost CARD well conserved to those of tetrapods; however, only 8 amino acid residues (Ile^25^, Leu^28^, Val^37^, Asp^56^, Gly^70^, Thr^93^, His^94^, and Ile^95^) in the cyprinid PYDs are conserved within the tetrapod CARDs. Furthermore, the 30th Asp (D) residue, related to the interaction of the CARD part of the ASC [49], is conserved in both tetrapod and general teleost CARDs, whereas this conservation does not exist in the cyprinid PYD (Figure S1). Consequently, the ASC-binding domain in the cyprinids showed a unique structure compared to those of the other vertebrates.

#### 2.2.2. Apoptosis-Associated Speck-Like Protein Containing a Caspase Recruitment Domain Formation

ASC is not only an inflammasome adaptor molecule; it also forms a speck, called ASC speck (also known as ASC pyroptosome), which is an oligomerized ASC that can also activate Casp1 [51,52,53,54]. In mammals, LPS or monosodium urete (MSU) induces ASC speck formation, then the ASC speck recruits and activates Casp1, induces IL-1β maturation, and triggers pyroptosis [52]. In zebrafish, ASC speck formation was induced by ASC overexpression or via the induction of inflammation with CuSO_4_ stimulation in vivo, after which the endogenous ASC recruits to the overexpressing ASC to form the speck [55]. Moreover, ASC overexpression induced speck formation in goldfish and turbot [43,47]. During overexpression of ASC, the PYD-deleted ASC demonstrated filament formation, and the CARD-deleted ASC diffused (not co-localized) into the cytosol of zebrafish and turbot [41,47]. In accordance with these results, the PYD domain of ASC could be the key to ASC speck formation in teleosts. The zebrafish ASC speck was co-localized with CaspA [41], suggesting that PYD-ASC interacts with PYD-CaspA (Figure 2).

### 2.3. The Mechanism of Pyroptosis via Caspase 1

#### 2.3.1. Induction of Pyroptosis through Caspase 1

Pro-Casp1 has two functional domains, an ASC-binding domain (CARD or PYD) and a caspase consensus (CASc) domain (Table 1). The CASc domain consists of p10 and p20 subunits, which are cleaved by self-proteolysis [56]. Then, the p10 and p20 subunits construct heterotetramers and play a role in converting pro-IL-1β, pro-IL18, or GSDMD to IL-1β, IL-18, or N-terminal GSDMD in mammals [22,57]. In seabass, pro-Casp1 can self-proteolyze into the active-form Casp1 (p10 and p20) while leaving a p24 fragment [58]. During PAMP and *Aeromonas hydrophila* stimulation, p10 and p20 subunits have been found in rainbow trout [59]. These results indicate that pro-Casp1 is self-proteolyzed into the active-form Casp1, consisting of the p10 and p20 subunits in teleosts and mammals.

#### 2.3.2. Induction of Pyroptosis in Fish

The N-terminus of GSDME is key in the induction of pyroptosis via its capabilities in pore formation on the cell membrane [60] (Figure 2). In the tongue sole, the CARD-deleted pro-Casp1 could cleave GSDME; thus, the N-terminus of GSDME induced pyroptosis through pore formation on the cell membrane [28]. In zebrafish, there are two types of GSDMEs (i.e., GSDMEa and GSDMEb), with a stronger induced pyroptotic cell death via GSDMEb-induced CaspB activation [27,29]. The mature forms of CaspB, which are associated with GSDME cleavage, occur in the presence of both p10 and p20 subunits [27]. In this case, the N-terminus of GSDME forms a pore on the cell membrane, which induces pyroptosis, and both the p10 and p20 subunits in the activated Casp1 are necessary for cleavage of GSDME in teleosts. In humans, Casp3 cleaves GSDME and induces pyroptosis [60]. Interestingly, in the WebLogo analysis, there is a consensus motif sequence “DMPD” at the Casp3 cleavage site of the mammalian GSDME, whereas teleosts have a different consensus motif sequence, “FEVD”, at the Casp1 cleavage site [28]. Casp1, Casp3, and Casp7 were recently shown to cleave GSDME in the tongue sole [28]. However, additional investigations for other factors that cleave GSDME and induce pyroptosis in teleosts are needed in the future.

#### 2.3.3. Induction of Pyroptosis via Non-Canonical Inflammasome Activation in Cyprinids

In mammals, Casp4/5/11 belong to the Casp1 family, and they directly recognize cytosolic LPS and activate it into mature forms to induce pyroptosis through GSDMD cleavage [54,60,61]. This inflammasome-independent induction of pyroptosis via Casp activation is called non-canonical inflammasome activation [61]. There are no existing orthologs of the Casp4/5/11 in most fish. However, CaspB in cyprinids shows activity similar to that of mammalian Casp5 [35,62]. The zebrafish CaspB shares the highest homology with that of human Casp5, and it also has a specific enzyme activity of the Casp5 substrate [30]. Besides, CaspB does not interact with the ASC speck [35]. During hemolysin-overexpressing *Edwardsiella piscicida* infection, Casp5 activity-dependent pyroptotic cell death was observed in zebrafish fibroblast cells [62]. The cells stimulated with LPS and cholera toxin B subunit (CTB), which transports LPS into the cell cytosol, also showed Casp5 activity and pyroptotic cell death [62]. Furthermore, the PYD domain of CaspB directly binds to LPS, and CaspB forms oligomers and induces pyroptosis via GSDMEb cleavage during hemolysin-overexpressing *E. piscicida* or cytosolic LPS stimulation [62] (Figure 2). In contrast, oligomerization and activation of CaspB did not result in the overexpression of CaspB alone [35]. Therefore, hemolysin from bacterial or cytosolic LPS stimulation may be necessary for non-canonical inflammasome activation in cyprinids.

## 3. Inflammasome-Related Gene Expression and Its Activation by Stimuli

An inflammasome activation model using a stimulant is important for understanding the inflammasome activation mechanism in detail. In mammals, NLRP3 inflammasome activation requires two steps. The first step is promoting the expression of NLRP3 and other inflammasome-related genes (called the priming step), and the second step is the formation and activation of the NLRP3 inflammasome [63]. A commonly used method of NLRP3 inflammasome priming is LPS stimulation, which recognizes TLR4 and promotes NF-κB-mediated gene expression of NLRP3 and inflammasome-related genes [63]. Moreover, NLRP3 needs to be phosphorylated by JNK1, located downstream of TLR signals for priming [63]. Several fish species, including Asian seabass, Japanese flounder, Japanese pufferfish, miiuy croaker, and rainbow trout, demonstrated that the NLR family or inflammasome-related genes were upregulated by LPS stimulation (Table 2) [39,46,64,65,66,67,68]. However, TLR4 does not exist in most fish, with it only located in cyprinid fish; moreover, TLR does not activate NF-κB via LPS stimulation in cyprinids [69,70]. Additionally, it is still unclear whether NLR family phosphorylation in fish is caused by LPS stimulation. Therefore, it is necessary to explore a suitable stimulant for inflammasome priming in fish. However, Pam3CSk4, a TLR2 ligand, is already used as a non-canonical inflammasome priming reagent in zebrafish [62]. In zebrafish, Pam3CSk4-stimulated TLR2 signaling was similar to that in mammals [71]. However, NLR family phosphorylation mechanisms are still unknown. Other than these stimulants, PAMPs (i.e., *Vibrio anguillarum* DNA [72], MDP [72], poly(I:C) [39,46,65,66,67,72], zymosan [39,46], flagellin [73], and peptidoglycan (PGN) [67,73]), and environmental factors (i.e., particulate silica [74] and cadmium [75]) may be used as stimulants for inflammasome priming in fish (Table 2). Thereafter, inflammasome activation in fish is indexed by the formation of the ASC speck, activation of Casp1, cleavage of IL-1β or GSDM, and the induction of pyroptosis (i.e., propidium iodide (PI) staining and measurement of LDH release from the cytosol), similar to that in mammals. Moreover, nigericin is the most common inflammasome activation inducer in mammals and induces inflammasome activation indices [76]. Nigericin is a *Streptomyces hygroscopicus*-derived antibiotic that acts as a potassium ionophore. Potassium efflux occurs in the cell, and this efflux activates the NLRP3 inflammasome in mammals [76]. In Japanese flounder, nigericin promotes Casp1 activity and IL-1β cleavage [36]. In several species of fish, nigericin induces the expression of inflammasome-related gene expression [42,43,68] (Table 2). Therefore, nigericin may induce the expression of inflammasome-related genes as stimulants in other fish species. Furthermore, extracellular ATP acts as an inflammasome activation inducer via the P2X7 receptor (P2X7R), a plasma membrane potassium ion channel, inducing potassium efflux in the cytosol [77,78,79,80]. P2X7R has been identified in several fish species, including Japanese flounder [81]. Here, nigericin and extracellular ATP promote Casp1 activation, IL-1β cleavage, and the induction of pyroptosis [65]. Moreover, Casp1 is activated by extracellular ATP in the orange-spotted grouper [44]. These results suggest that inflammasome activation may be promoted by potassium efflux in fish as well as in mammals via nigericin or extracellular ATP factors. The inflammasome-related genes were upregulated with extracellular ATP stimulation in several species of fish [39,44,46] (Table 2). Thus, extracellular ATP may promote inflammasome activation as well as priming in fish. However, it is unclear whether potassium efflux occurs with nigericin or extracellular ATP stimulation, and which receptors sense this efflux in fish remains unclear. Therefore, it is necessary to further understand the relationship between the inflammasomes and potassium efflux in fish.

## 4. Inflammasome Activation during Pathogenic Infection

Inflammasome activation helps to restrict pathogen replication, including in bacteria and viruses [19]. In mice, NLRP1B inflammasome activation plays a crucial role in the host’s defense against infection with the Gram-positive bacteria *Bacillus anthracis* [90] and the intracellular parasite *Toxoplasma gondii* [31]. The NLRP3 inflammasome is activated by a wide range of pathogens, including Gram-positive and Gram-negative bacteria (e.g., *Staphylococcus aureus* [91], *Mycobacterium tuberculosis* [92,93,94], *E. tarda* [95,96,97], *Listeria monocytogenes* [98]) [99], RNA and DNA viruses (e.g., influenza virus, adenovirus [100], SARS-CoV-2 [101]) [102], fungi (e.g., *Candida albicans* [103]), and parasites (e.g., *Leishmania amazonensis* [104]) in mammals. The relationship between the inflammasomes and *E. piscicida* (also known as *E. tarda* in mammals) and *Aeromonas* sp. infection have been previously investigated in mammals and teleosts.

*Edwardsiella piscicida* is an intracellular Gram-negative bacterium that causes edwardsiellosis [105]. In mammals, *E. piscicida* infection alone does not cause an inflammasome priming step. However, zebrafish *nlrp1* and *nlrp3* gene expression was induced by *E. tarda* [27,35], and the expression of the *nlrp3* gene in Japanese flounder was upregulated during *E. piscicida* infection [27] (Table 2). Therefore, it is inferred that *E. piscicida* infection causes the inflammasome priming step in teleosts, unlike in mammals. The type III secretion system (T3SS) of *E. piscicida* induced both ASC and Casp1-dependent NLRP3 and NLRC4 inflammasome activation (i.e., Casp1 activation, IL-1β secretion, and pyroptosis) in mouse bone marrow-derived macrophages (BMDMs). However, the LPS-priming step was necessary for activation [95,97]. The *E. piscicida* virulence effector trxlp also activated the NLRC4 inflammasome in mouse BMDMs and the mouse macrophage-like cell line J774A.1 [96]. However, the *E. piscicida* type VI secretion system (T6SS) effector protein (EvpP) suppressed NLRP3 inflammasome activation via ASC oligomerization by inhibiting calcium ion-dependent JNK (c-Jun N-terminal kinase) signals in the J774A.1 cells [97]. Furthermore, EvpP inhibited NLRC4-mediated bacterial clearance in the mouse spleen and liver [97]. Additionally, the T3SS of *E. piscicida* promoted intracellular invasion and pyroptosis, and it suppressed bacterial replication in human epithelial cells, such as the HeLa cell line [106]. In Japanese flounder head kidney macrophages, *E. piscicida* T3SS promoted Casp1 activity, IL-1β maturation, and pyroptosis induction [36]. Conversely, EvpP suppressed inflammasome activation by inhibiting JNK signaling in zebrafish larvae; thus, *E. piscicida* T6SS contributed to the colonization of zebrafish larvae [84,106]. Moreover, the mortality rate during wild type *E. piscicida* infection is higher than those of either T3SS or T6SS deficient strains [106]. In zebrafish liver epithelial or fibroblast cells, hemolysin (EthA^+^)-overexpressing *E. piscicida* 0909I strains induced CaspB-dependent pyroptotic cell death [62]. Thus, *E. piscicida* virulence factors, especially T3SS, T6SS, and hemolysin, are related to fish canonical and non-canonical inflammasome activation. In teleosts, *E. piscicida* pathogenicity depends on both T3SS and T6SS. Furthermore, due to the relationship between the inflammasome and *E. piscicida* infection, it has been considered that the intracellular bacteria can proliferate intracellularly, employing a mechanism to suppress inflammasome activation and pyroptosis, such as in the case of *E. piscicida* T6SS. Clarifying the relationship between the fish inflammasome and *E. piscicida* T3SS and T6SS may lead to the future suppression of edwardsiellosis in aquaculture.

*Aeromonas* spp., such as *A. hydrophila* and *A. veronii*, are Gram-negative bacteria that cause gastroenteritis in mammals and act as opportunistic infectious diseases in fish. In mouse BMDMs, *A. veronii* aerolysin, a type of *Aeromonas* sp. hemolysin, and T3SS induced NLRP3-dependent Casp1 activation and pyroptosis [107]. Furthermore, *A. hydrophila* T3SS induced ASC-dependent NLRP3 and NLRC4 inflammasome activation (i.e., Casp1 activation, IL-1β cleavage, and pyroptosis) in BMDMs [108]. In teleosts, the gene expression of the striped murrel *casp1* and Japanese medaka *asc1* was upregulated during *A. hydrophila* infection [42,88]. In rainbow trout, Casp1 activity was promoted during LPS or zymosan priming after *A. hydrophila* infection [59]. In a recent study, pyroptotic-like cell death was promoted by *A. hydrophila* infection in Japanese medaka kidney cells; however, cell death was suppressed in ASC knockout (KO) medaka kidney cells [109]. Moreover, *A. hydrophila* burden was observed in the ASC-KO medaka compared to the wild type [109]. These results suggest that the inflammasome is probably primed and activated during *A. hydrophila* infection and inflammasome activity plays an important role in eliminating *A. hydrophila* in teleosts. Other than these pathogens, as well as bacterial infection (i.e., *V. anguillarum* [72], *Bacillus subtilis* [83], *E. ictaluri* [110], *V. alginolyticus* [67], *S. aureus* [67], *Lactobacillus paracasei* spp. *paracasei* [68], *Aphanomyces invadans* [88], *Streptococcus iniae* [88], and *S. agalactiae* [40]), viral infection (rhabdovirus spring viremia of carp virus (SVCV) [21]) can also induce the expression of inflammasome-related genes, Moreover, *Salmonella enterica* sv. Typhimurium [82] or *Francisella noatunensis* [85] promoted Casp1/CaspA activity in teleosts (Table 2).

## 5. Conclusions

In the last decade, the structure of inflammasomes, as well as the mechanism and role of inflammasome activation and pyroptosis during pathogenic infections, have been elucidated in mammals. Inflammasome and pyroptosis-related genes, such as the NLR family members (e.g., *nlrp1*, *nlrp3*), *asc*, *casp1*, and *gsdme*, have been identified in numerous fish species, and the functions of the molecules they encode are becoming clear. However, there are very few studies on inflammasome activation via these molecules in teleosts. This is because there may be multiple molecules with different structures or activities, depending on the fish species selected. It is also different among small fish model organisms, such as the differences between zebrafish and Japanese medaka (Figure 2). In particular, the structure of pro-Casp-1, which is the inflammasome effector molecule, differs between most fish and cyprinids. Furthermore, there is non-canonical inflammasome activation in the cyprinid, whereas there are no reports of this occurring in other fish species. Therefore, it is necessary to proceed with research, keeping in mind that it depends on the fish species. During bacterial infection, inflammasome activation may restrict pathogen replication in teleosts and mammals. A better understanding of the relationship between inflammasomes and pathogens in teleosts will lead to the development of new immunostimulants to suppress aquaculture diseases.

## Figures and Tables

**Table 1:** Structures of typical and cyprinid-types pro-Caspase 1 in vertebrates.

	Species	Formation Type	Gene Name	Ensembl Gene ID	Domain Structure
Mammal	Human (*Homo sapiens*)	Typical-type	*CASP1*	ENSG00000137752	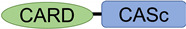
	Mouse (*Mus musculus*)	Typical-type	*Casp1*	ENSMUSG00000025888	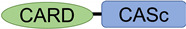
Bird	Chicken (*Gallus gallus*)	Typical-type	*CASP1*	ENSGALG00000001049	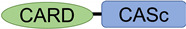
Reptile	Common wall lizard (*Podarcis muralis*)	Typical-type	*CASP1*	ENSPMRG00000020733	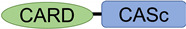
Amphibian	Tropical clawed frog (*Xenopus tropicalis*)	Typical-type	*casp1*	ENSXETG00000007792	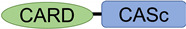
Fish	Japanese medaka (*Oryzias latipes)*	Typical-type	*casp1*	ENSORLG00000006320	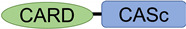
	Japanese pufferfish (*Takifugu rubripes*)	Typical-type	*casp1*	ENSTRUG00000007971	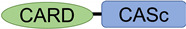
	Gilthead seabream (*Sparus aurata*)	Typical-type	*casp1*	ENSSAUG00010008488	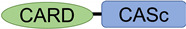
	Turbot (*Scophtalmus maximus*)	Typical-type	*casp1*	ENSSMAG00000013017	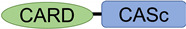
	Zebrafish (*Danio rerio*)	Cyprinid-type	*caspa*	ENSDARG00000008165	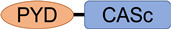
			*caspb*	ENSDARG00000052039	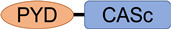
			*caspbl*	ENSDARG00000094433	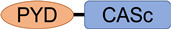
	Common carp (*Cyprinus carpio*)	Cyprinid-type	*caspa*	ENSCCRG00000035668	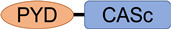
			*caspb*	ENSCCRG00000040063	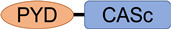

The pro-Casp1 consists of CARD as an ASC-binding domain and CASc as the mature form of Casp1 in tetrapods (i.e., mammals, birds, reptiles, and amphibians) and fish except for cyprinid fish. In contrast, only cyprinid fish species possess the pro-Casp1, including PYD and CASc, instead of CARD. Furthermore, the cyprinid fish have multiple Casp1 orthologs, whereas the other vertebrates have a single Casp1 gene.

**Table 2:** Inflammasome priming and activation by pathogenic infection or stimulation in fish.

Species	Target	Infection Models	Stimulation Models	Reference
Gilthead seabream (*Sparus aurata*)	*casp1* gene	*Vibrio anguillarum* *Bacillus subtilis*	*Vibrio anguillarum* DNA MDP	[72,82,83]
	Casp1 activity	*Salmonella enterica* sv. Typhimurium	osmotic pressure	
Japanese flounder (*Paralichthys olivaceus*)	*asc* gene	*Edwardsiella tarda*	LPS poly (I:C) zymosan extracellular ATP	[36,39,46,64,65]
	*casp1* gene	*E*. *tarda* *E. piscicida*	LPS poly (I:C) extracellular ATP	
	*nlrb* gene	*E*. *ictaluri*		
	*nlrc* gene	*E*. *ictaluri*	LPS	
	*nlrc3* gene	*E*. *tarda*	LPS poly (I:C) extracellular ATP	
	*casp1* and *nlrp3* genes	*E. piscicida*		
	Casp1 activity	*E. piscicida* (EIB202)	extracellular ATP nigericin MSU	
Zebrafish (*Danio rerio*)	*asc* and *caspa* genes	Rhabdovirus spring ciremia of carp virus (SVCV)*E*. *tarda*		[21,27,29,35,62,84,85]
	*nlrc3l1* gene	*E. piscicida*		
	*nlrp1*, *nlrp3,* and *caspb* genes	*E*. *tarda*		
	CaspyA activity	*Francisella noatunensis* *E*. *piscicida_ΔevpP*	H_2_O_2_	
	CaspyB activity	*E*. *piscicida* (0909I)	CTB+LPS	
Miiuy croaker (*Miichthys miiuy*)	*nlrc3* gene	*V*. *anguillarum*	poly (I:C)	[86,87]
	*nlrc35, nlrc39,* and *nlrc40* genes	*V*. *anguillarum*	LPS poly (I:C)	
Rainbow trout (*Oncorhynchus mykiss*)	*nlrc3* gene		LPS poly (I:C)	[59,66]
	Casp1 activity	LPS or zymosan + *A. hydrophila*		
Asian seabass (*Lates calcarifer*)	*nlrc3* gene	*V*. *alginolyticus* *Streptococcus aureus*	LPS poly (I:C) PGN	[67]
Goldfish (*Carassius auratus*)	*asc* gene		nigericin	[43]
	*nlrc3l* gene		ATP	
Japanese pufferfish (*Takifugu rubripes*)	*nlr-c1*-*8* and *10*-*12* genes	*Lactobacillus paracasei* spp. *paracasei*		[68,74]
	*nlr-c1*-*5*, *7*-*10*, *12* and *13* genes		LPS	
	*nlr-c1*, *5*, *7*, *10* and *12* genes		nigericin nigericin+LPS	
	*asc* and *casp1* genes		particulate silica	
	*nlrc5*-*13*, *nlrc3* genes		particulate silica	
Striped murrel (*Channa striata*)	*casp1* gene	*Aphanomyces invadans* *Aeromonas hydrophila*		[88]
Tongue sole (*Cynoglossus semilaevis*)	*casp1* gene	*E*. *tarda*		[89]
Turbot (*Scophtalmus maximus*)	*asc* gene	*E*. *piscicida*		[47]
	*nlrc3a* and *nlrc3b* genes	*V*. *anguillarum* *St*. *iniae*		
Common carp (*Cyprinus carpio*)	*casp1* and *nlrp3* genes		cadmium	[73,75]
	*nlrc* gene	*V*. *anguillarum*	poly (I:C) flagellin PGN	
Nile tilapia (*Oreochromis niloticus*)	*nlrc3* gene	*St*. *agalactiae*		[40]
Orange-spotted grouper (*Epinephelus coioides*)	*asc* and *casp1* genes		ATP	[44]
	Casp1 activity		ATP	
Japanese medaka (*Oryzias latipes*)	*asc1* gene	*Ae*. *hydrophila*		[42]
	*asc2* gene		nigericin	

## Data Availability

Publicly available datasets were analyzed in this study. This data can be found here: https://www.ncbi.nlm.nih.gov (accessed on 21 April 2021).

## Supplementary Materials

The following are available online at https://www.mdpi.com/article/10.3390/ijms22094389/s1, Figure S1: Comparison of the caspase recruitment domain (CARD) and pyrin domain (PYD) of the pro-Caspase1 (Casp1) amino acid sequences.
