# Supplementary material for: Inflammasomes in Teleosts: Structures and Mechanisms That Induce Pyroptosis during Bacterial Infection
Source: Int J Mol Sci. 2021 Apr 22;22(9):4389. doi: 10.3390/ijms22094389 (PMC8122782; doi:10.3390/ijms22094389)
Supplement: Supplementary file 1 [file ijms-22-04389-s001.zip › ijms-1182325-supplementary.pdf]

Supplementary Material

Inflammasomes in Teleosts: Structures and Mechanisms That Induce Pyroptosis during Bacterial Infection

Natsuki Morimoto [1], Tomoya Kono [2], Masahiro Sakai [2] and Jun-ichi Hikima [2,*]

[1] Interdisciplinary Graduate School of Agriculture and Engineering, University of Miyazaki, 1-1 Gakuenkibanadai-nishi, Miyazaki 889-2192, Japan; nmorimoto0902@gmail.com

[2] Department of Biochemistry and Applied Bioscience, Faculty of Agriculture, University of Miyazaki, 1-1 Gakuenkibanadai-nishi, Miyazaki 889-2192, Japan; tkono@cc.miyazaki-u.ac.jp (T.K.); m.sakai@cc.miyazaki-u.ac.jp (M.S.)

* Correspondence: jhikima@cc.miyazaki-u.ac.jp; Tel./Fax: +81-985-58-7230

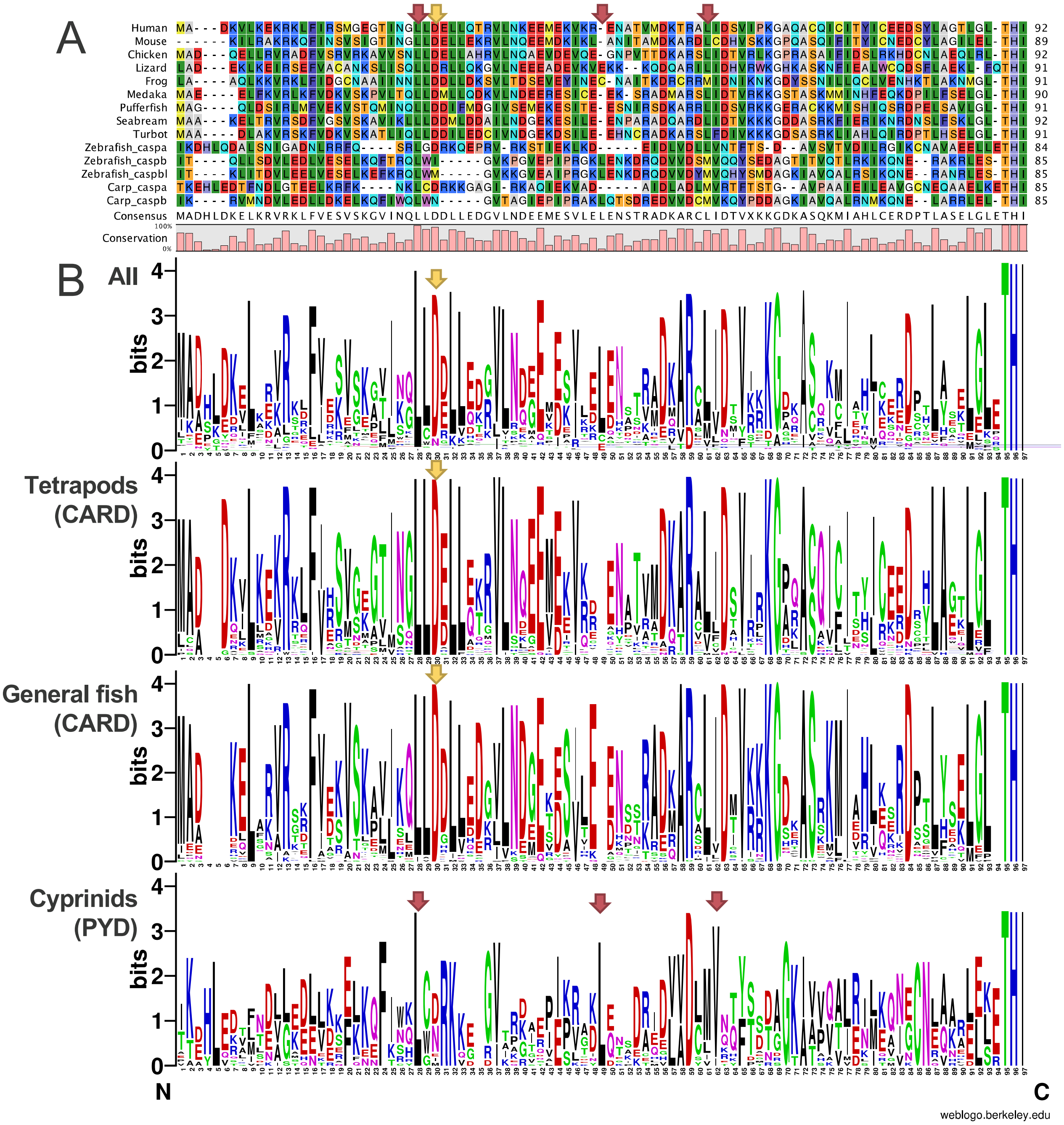

Figure S1. Sequence comparison of the CARD or PYD of the pro-Casp1. Multiple sequence alignment (A) and Weblogo analysis (B) of the CARD or PYD of the pro-Casp1 from tetrapods, general fish, and cyprinids. The amino acid sequences obtained from Ensembl genome database (https://www.ensembl.org) were aligned with CLC Sequence viewer v7.7.1 (Quigen), and the WebLogo analysis was performed using WebLogo program v2.8.2 (https://weblogo.berkeley.edu). In the WebLogo analysis, the amino acid sequences of 39 tetrapods (Ensembl transcript ID: ENST00000533400.6, ENSMUST00000027015.7, ENSBBBT00000024946.1, ENSXETT00000017024.4, ENSACAT00000013632.2, ENSCDRT00005002821.1, ENSSBOT00000026622.1, ENSCCAT00000030142.1, ENSPVIT00000015190.1, ENSGALT00000001571.6, ENSNPET00000004469.1, ENSPTRT00000086615.1, ENSCGRT00015002981.1, ENSBGRT00000038384.1, ENSMLET00000037831.1, ENSMLET00000046544.1, ENSNLET00000049341.1, ENSACCT00020014051.1, ENSGEVT00005018053.1, ENSGGOT00000058923.1, ENSECAT00000035835.2, ENSMMUT00000106726.1, ENSCJAT00000019123.4, ENSHGLT00100003002.1, ENSMMNT00015022840.1, ENSARWT00000010168.1, ENSPANT00000058585.1, ENSPANT00000017402.2, ENSMODT00000035608.3, ENSSSCT00000076386.1, ENSMNET00000007897.1, ENSOANT00000016594.4, ENSOCUT00000001102.4, ENSVVUT00000024917.1, ENSCPUT00000016822.1, ENSCATT00000038205.1, ENSXETT00000089520.1, ENSPSNT00000016214.1, ENSCHYT00000000313.1), 51 general fish (Ensembl transcript ID: ENSSFOT00015005791.2, ENSGMOT00000072973.1, ENSSSAT00000112480.1, ENSSPAT00000012245.1, ENSSTUT00000049240.1, ENSHBUT00000014379.1, ENSHBUT00000014356.1, ENSCGOT00000026382.1, ENSOTST00005081780.1, ENSATET00000020757.2, ENSATET00000020789.2, ENSAOCT00000005938.1, ENSOKIT00005103268.1, ENSACLT00000025736.1, ENSACLT00000025757.1, ENSTRUT00000092238.1, ENSSAUT00010019886.1, ENSSDUT00000026719.1, ENSPRET00000002390.1, ENSOMET00000032149.1, ENSORLT00000007939.2, ENSSFAT00005003178.1, ENSENLT00000016855.1, ENSNBRT00000007017.1, ENSNBRT00000006974.1, ENSPNYT00000007387.1, ENSPNYT00000007612.1, ENSFHET00000022661.1, ENSONIT00000004810.2, ENSSORT00005038871.1, ENSSORT00005038884.1, ENSSLUT00000019324.1, ENSXMAT00000024215.1, ENSXMAT00000018545.2, ENSXMAT00000018549.2, ENSPLAT00000006713.1, ENSPLAT00000006584.1, ENSPLAT00000006706.1, ENSCVAT00000010283.1, ENSCVAT00000010345.1, ENSPMET00000004603.1, ENSBSLT00000005028.1, ENSAPOT00000034889.1, ENSLOCT00000001662.1, ENSHCOT00000008910.1, ENSHCOT00000009192.1, ENSSMAT00000021490.1, ENSSLDT00000026936.1, ENSMZET00005022651.1, ENSMZET00005022657.1, and ENSMAMT00000018611.2), and 16 cyprinids (Ensembl transcript ID: ENSSGRT00000034028.1, ENSSGRT00000012961.1, ENSSGRT00000037551.1, ENSCART00000060655.1, ENSCART00000018761.1, ENSCART00000000221.1, ENSCART00000093702.1, ENSSRHT00000064810.1, ENSSRHT00000025250.1, ENSSRHT00000000282.1, ENSPNAT00000023155.1, ENSCCRT00000071734.1, and ENSCCRT00000080458.1) were used. The yellow arrows show the ASC-binding residues (30th Asp), and the red arrows show the hydrophobic residues, which may interact with ASC via PYD–PYD interactions.
